# On East-Indian Opium

**Published:** 1829-06

**Authors:** John Webster

**Affiliations:** Physician to the St. George's and St. James's Dispensary, &c.


### EAST-INDIAN OPIUM.

*On East-Indian Opium.* By John Webster, m.d. Physician to the St. George's and St. James's Dispensary, &c.

At a recent meeting of the Westminster Medical Society, specimens of East-Indian opium were exhibited to the members, superior, it is believed, to any which has hitherto been met with in this country; but, as it was almost impossible, at a public meeting, to give a full or accurate description of so valuable a drug, more especially as the hour for adjourning the Society had nearly elapsed, the following additional observations are subjoined; which, although brief, may not be altogether uninteresting. Should they tend to direct the attention of medical men to further and more particular investigation, it will be gratifying, and must also prove very beneficial both in a commercial and scientific point of view, could the supply of opium for the home, and it may be said for the European market, always be obtained from a country whose prosperity is so intimately connected with England as the East Indies undoubtedly is; for surely every one must allow that such commercial transactions would be far preferable to continuing, as at present, dependent for this indispensable medicine upon the good will of a nation like the Turkish, who, although considered as "an ancient ally," have, however, never been celebrated for liberality towards this or any other Christian nation.

I lately received a specimen of East-Indian opium from my friend, Dr. Adam, secretary to the Medical Board at Calcutta, along with a letter containing the following account:

"I embrace the opportunity of my friend, Mr. ———, proceeding to England, to send you a sample of very pure opium, prepared for medicinal purposes under the direction of our Board, and which I should hope will be found superior to any Turkish in the home market. I am disposed to think that such a preparation as this, sent home in fixed quantities, and bearing the stamp of the Honourable Company as a voucher for its purity, would soon come into general use, and supersede all other varieties of the drug met with. Captain Jeremie, who manufactures it, is an assistant to the opium agents, and has already done much to improve the quality of the opium generally prepared in the Bihar division. He very ingeniously has contrived many modes of packing; but this, in plates of mica, and placed in a strong teakwood box, appears to me preferable to all others."

In compliance with the request of Dr. Adam, and being also anxious to ascertain, from personal experience, the strength and properties of an opium so much recommended, many trials have been made, particularly at the St. George's and St. James's Dispensary, as also by several medical practitioners, to whom portions of the drug had been furnished; and, whether given in substance or in the form of tincture, it appeared to be quite equal to any Turkey opium. One gentleman, indeed, considered it superior, in so far that those unpleasant feelings in the head did not so frequently follow its exhibition as after opium of the ordinary kind. From repeated observations, it is therefore considered that this variety of opium may advantageously be substituted for the Turkish, especially since it was stated at the Westminster Medical Society, that the home market may be supplied with East-Indian opium on much lower terms than with the other. Should subsequent experience, therefore, confirm what is now advanced, there can be little doubt but it will ultimately come into general use.

In appearance, this East-Indian opium very much resembles socotorine aloes, both in weight and texture, being only a little darker in the colour and with a slight tinge of red: it is consequently very unlike any of the same medicine hitherto imported, which has usually been of the consistence of common tar, and of not more than half the strength of that brought from the Levant. In taste and smell it is very similar to the Turkish, though perhaps not quite so powerful, excepting when heated. If made into tincture, the preparation is of a beautiful dark colour, and perfectly clear; and, as almost no residuum remains in the vessel in which it is made, scarcely any loss, either of the spirit or the opium, is sustained; which, it is well known, forms a very considerable objection, in point of expense, to the tincture usually employed.

As it has generally been supposed that the efficacy of opium especially depends upon the quantity of morphia it contains, portions of the East-Indian variety were subjected to analyzation, in order to ascertain the proportion of that, as well as of the other ingredients; and Dr. Wm. Gregory, assistant to Dr. Turner, professor of chemistry in the University of London, having kindly undertaken the task, the following statement has been drawn up, as the result of his investigation:

"The specimen of East-Indian opium which you gave me weighed about four hundred grains, after being dried at a gentle heat. From this quantity I obtained fifteen grains of morphia, somewhat coloured, but quite crystalline. Operating on so small a quantity, however, it is impossible to avoid considerable loss; nor could I ascertain the amount of any of the other ingredients. The opium is quite free from extraneous matter: it does not, however, seem to contain less colouring or extractive matters than Turkish opium. It appears to me to contain also narcotine; but, without a separate process, this substance, the quantity of which is small, is with difficulty extracted. The morphia is, as in common opium, combined with meconic acid, which was easily detected by its power of reddening the solutions of peroxide of iron. I should think the quantity of morphia as great as in good Turkish opium, if not greater; that is, about five drachms in the pound of opium; but I do not see any reason for supposing the opium to be more efficacious in practice. If it can be brought into the market at a cheaper rate, it will probably supplant the present kinds, especially from its being so clean and free from adulteration."

"To Dr. Webster."

Dr. A. T. Thomson, professor of materia medica in the University of London, also most kindly volunteered to analyze a portion of the East-Indian opium; and, although the result shows a smaller proportion of morphia than Dr. W. Gregory obtained, it is still highly important and satisfactory. The following is Dr. Thomson's letter:

"The specimen of East-Indian opium which you gave me possesses many advantages over all the other specimens of that variety of opium that have come under my observation: it is cleaner, has a higher narcotic odour, and is more soluble in water at the temperature of 60° Fahrenheit than the ordinary Indian opium. One disadvantage, at least as far as relates to its analysis, or as forming a source of the salts of morphia, is the large quantity of colouring matter which it contains. It was submitted to the following analysis: Four hundred and eighty grains were rubbed up with three pints of distilled water, and macerated for twenty-four hours; the solution was then filtered, and the meconiate of morphia contained in it decomposed by ammonia, which threw down the morphia, in combination with the colouring and some gelatinous matter. This precipitate, well washed, was mixed with a few ounces of distilled water, and converted into an acetate by the addition of pure acetic acid; it was next freed from the colouring matter, by mixing it with animal charcoal deprived of its salts; and, the solution being filtered, was again decomposed by ammonia, added in excess. The morphia thus precipitated was first washed with weak spirit, in order to free it as completely as possible from colouring matter, and then treated with strong boiling alcohol. I send you the result. The crystals are not so white as they would be, were they dissolved again and recrystallized; but I was anxious to lose as little of the product as possible. I have not weighed the salt; and, in leaving this to you, I think you may allow three or four grains for loss.

"I am of opinion that opium, prepared in India with the care that has evidently been bestowed upon this specimen, cannot fail to find a ready market in Europe. It is not likely to surpass the opium prepared in Persia, the Turkish opium of the European market; but, if it never can attain to the same degree of excellence, on account of climate, still it will be a valuable article, if it come near it in quality."

"To Dr. Webster."

The quantity of morphia obtained by the above process from the 480 grains of opium weighed exactly six grains, to which are to be added four grains for loss; thus being about one third less than the product obtained by the other analysis.

As it is proposed still further to investigate the nature and properties of East-Indian opium, more especially in reference to its utility as a remedy in the cure of disease, another report may perhaps be given, should any facts worth communicating present themselves.

*56, Grosvenor street; 11th May,* 1829.

---

